# Fear, anxiety, and knowledge levels of women who underwent gynecologic surgery during the COVID-19 pandemic

**DOI:** 10.1590/1806-9282.20240422

**Published:** 2024-09-30

**Authors:** Mehmet Ferdi Kıncı, İlknur Yeşilçınar, Gülten Güvenç, Hikmet Can Ünver, Ahmet Akın Sivaslıoğlu

**Affiliations:** 1 İzmir Bayraklı City Hospital, Department of Obstetrics and Gynecology – İzmir, Turkey.; 2 Izmir Katip Celebi University, Faculty of Health Sciences, Department of Nursing – İzmir, Turkey.; 3 University of Health Sciences, Gülhane Faculty of Nursing – Ankara, Turkey.; 4 Muğla Sıtkı Koçman University, Educational Research Hospital, Department of Obstetrics and Gynecology – Muğla, Turkey.; 5 Izmir University of Economics, Medical Point Hospital, Department of Obstetrics and Gynecology – İzmir, Turkey.

**Keywords:** COVID-19, Gynecology, Surgery, Anxiety, Fear, Knowledge

## Abstract

**OBJECTIVE:**

The objective of this study was to evaluate the fear, anxiety, and knowledge level in women who underwent gynecological surgical procedures during the COVID-19 pandemic.

**METHODS:**

This cross-sectional study was conducted on 188 women who underwent a gynecologic surgical operation in Muğla, Turkey. Data were collected by using demographics and obstetric detail form, questionnaire on knowledge, attitudes, and practice toward COVID-19, and State-Trait Anxiety Inventory-I (STAI-I).

**RESULTS:**

Most of the women perceived their surgical process as very urgent. Women felt fear mostly for being infected with the virus, and they were afraid of transmitting COVID-19 to another one. The COVID-19 knowledge scores of women who had undergone cancer surgery were statistically significantly higher than others (p=0.017). The STAI-I scores of women increased as their COVID-19 knowledge scores increased (p<0.05).

**CONCLUSION:**

This study demonstrated that women were afraid of COVID-19 infection during gynecological operation and due to hospitalization, sociodemographic characteristics affected the knowledge levels about COVID-19 infection, and the anxiety levels of the women. Planning appropriate interventions to decrease the fear and anxiety of women who undergo gynecological surgery during the pandemic is important to ensure that women adhere to their treatment and follow-up in the postoperative period.

## INTRODUCTION

The COVID-19 pandemic has interrupted the provision of healthcare services, resulting in urgent and mandatory changes^1^. In 2020, it was reported that nearly 40% of adults delayed or avoided medical care during the pandemic due to concerns about COVID-19 in the United States (US)^2^. The Royal College of Obstetricians and Gynaecologists and the British Society for Gynaecological Endoscopy recommend the active use of nonsurgical techniques if possible and the postponement of elective gynecological operations in order to reduce the number of admissions^3^. It is estimated that around 28 million surgeries have been postponed or canceled worldwide due to the pandemic^4,5,6,7^.

In addition to restricting the number of surgical operations during the pandemic, patients are afraid of COVID-19 infection during surgical procedures. Women with gynecological problems have postponed their hospital visits or have not presented to the hospital for fear of being infected with COVID-19. It has been reported that this causes more negative effects, especially on women who require vital surgeries, such as gynecological cancer surgery^5,7-10^. During this period, patients were also affected psychologically. Although various measures have been attempted to be taken to oblige patients to have PCR tests before surgical procedures, to encourage the use of personal protective equipment, and to prevent possible COVID-19 transmission by restricting visitors, these interventions may not be sufficient to relieve patients’ concerns^6^. Therefore, this study aimed to evaluate the fear, anxiety, depression, and knowledge level in women who underwent gynecological surgical procedures during the COVID-19 pandemic.

## METHODS

### Design

This cross-sectional study was conducted in a training and research hospital in Muğla, Turkey, between May and August 30, 2021. The study included women who underwent a gynecologic surgical operation using laparoscopy or laparotomy techniques, at least primary school graduates, and volunteers.

### Sample size and participants

The sample size was determined using an online sample size calculator^11^. The total gynecologic surgery number of the previous year (2020) of the gynecology clinic was 600 women. The sample size was determined as 188 women (confidence level=90%, margin of error=5%, population proportion=50% and population size=600, α=0.05, and power=80%).

### Data collection

Research data were collected on the first postoperative day after gynecologic surgery through face-to-face interviews. Women’s written and verbal consent was obtained.

### Data collection forms

#### Demographics and obstetric detail form

This form consists of three parts. The first part consists of 14 questions about demographics such as age, educational status, and so on. The second part consists of seven questions. Two of them were about the surgical procedure that women underwent. Five of them addressed perceived risk and one question about COVID-19 testing status. The third part of the form consists of two questions regarding COVID-19-related fear and two questions about the surgical procedure. A visual analog scale (VAS-10-cm horizontal scale) was used to determine perceptions of fear of COVID-19 transmission.

#### Questionnaire on knowledge, attitudes, and practice toward COVID-19

The questionnaire consists of 12 questions: 4 regarding clinical presentations, 3 regarding transmission routes, and 5 regarding prevention and control of COVID-19^12^. The response is to be selected among the options of “true,” “false,” or “I don’t know.” The correct answer graded as one point, and the highest possible score is 12. Cronbach’s alpha in the original study was 0.71^12^ and was 0.70 in our study.

#### State-Trait Anxiety Inventory-I

This 4-point Likert-type scale consists of 20 items. The total score ranges between 20 and 80 points, with a higher score denoting a higher anxiety level^13,14^. The Cronbach’s alpha value of the scale was found as 0.83^14^, and it was 0.70 in our study.

### Data analysis

The IBM SPSS Statistics 26 software was used for analysis. For descriptive statistics, numbers, and percentages, arithmetic means and standard deviation were used. The normality of the numeric variables was tested using the Kolmogorov-Smirnov test. For the statistical comparison of the descriptive data, one-way analysis of variance (ANOVA), Pearson correlation, Tukey test, and t-test were used. The relationships between scale scores were calculated with Pearson’s correlation coefficient. A p<0.05 value was accepted as statistically significant.

### Ethical considerations

Ethical approval was granted from the Muğla Sıtkı Koçman Education and Training Hospital ethical committee (Number: 200305/88, Date: 28.04.2021).

## RESULTS

Women’s demographic data are shown in Table 1. According to the data not shown in the tables, of the women, 83.0% stated that they experienced fear of coronavirus transmission, and 58.0% stated that the pandemic partially affected their mental health. The most common concerns regarding the surgical process were (57.4%) being infected with the virus and uncertainty (36.7%). The majority of the women perceived their surgical process as very urgent. Women felt fear mostly for being infected with the virus (67.6%), and they were afraid of transmitting COVID-19 to another one (47.9%). They were also concerned with their family’s health (44.7%) was the other most common factor affecting women during the COVID-19 pandemic.

**Table 1:** Social and demographic characteristics of the women.

Characteristics	Group (n=188)	
	Mean	±SD
Age	49.47	10.56
	**n**	**%**
Marital status		
Single	33	18.6
Married	155	81.4
Educational status		
Illiterate	10	5.3
Primary school	126	67.1
High school	27	14.4
University and higher	25	13.4
Employment status		
Employed	35	18.6
Unemployed	147	78.2
Retired	6	3.2
Menopause status		
Natural	76	40.4
Surgical	38	20.2
No menopause	74	39.4
Income status		
Income less than expenses	63	33.5
Equal income and expense	103	54.8
Income more than expenses	22	11.7

SD: standard deviation.

Married women had statistically significantly higher STAI-I scores than single women (p=0.037). The COVID-19 knowledge scores of working and non-working women were higher than retired women (p<0.001). The anxiety scores of primary school graduate women were higher than those with other educational levels (p=0.037). Women who paid attention to warnings about COVID-19 had higher anxiety scores (p=0.015). The COVID-19 knowledge scores of women who had undergone cancer surgery were statistically significantly higher than others (p=0.017). The anxiety levels of women who perceived the urgency of the surgical operation to be very urgent were statistically significantly higher (p=0.011). Women’s level of knowledge who stated that COVID-19 did not affect their mental health was statistically significantly higher (p=0.017) (Table 2). The STAI scores of women increased as their COVID-19 knowledge scores increased (Table 3). According to the data not shown in the tables, women’s type of gynecological operations did not affect their STAI and COVID-19 knowledge scores.

**Table 2:** Average scores of participants on the COVID-19 Knowledge Scale and State-Trait Anxiety Inventory-I according to their socio-demographic and COVID-19-related characteristics.

Characteristics		COVID-19 Knowledge Scale				STAI-I			
		Mean	±SD	Z	p	Mean	±SD	Z	p
Marital status	Single	2.32	0.90	-1.422	0.155	42.33	8.99	-2.081	0.037^a^
	Married	2.77	1.68			45.52	8.29		
Employment status	Yes	3.57	2.11	19.755	<0.001^b^	46.71	7.96	2.711	0.258
	No	2.44	1.36			44.81	8.47		
	Retired	3.33	0.51			38.33	9.64		
Education	Illiterate	2.40	0.84	0.770	0.857	41.40	7.38	8.483	0.037^b^
	Primary school	2.72	1.83			46.05	7.86		
	High school	2.59	1.00			42.92	8.39		
	University and higher	2.76	0.92			43.08	11.07		
Paying attention to warnings about COVID-19	Yes	2.44	1.13	-0.485	0.628	52.30	8.74	-2.437	0.015^a^
	Somehow	2.73	1.58			44.52	8.35		
Fear of COVID-19	Yes	2.52	1.16	-1.675	0.094	45.33	8.13	-1.298	0.194
	No	3.71	2.76			42.80	10.23		
Diagnosis	Myoma, polyp	2.55	1.57	10.180	0.017^b^	45.03	8.73	1.381	0.710
	Ovarian cyst	2.22	0.88			44.38	7.47		
	CA	3.41	2.14			45.05	8.79		
	Prolapse	2.81	0.95			45.00	8.81		
Perceived urgency of surgical procedure	Urgent	3.00	1.15	0.719	0.396	52.00	8.08	6.547	0.011^b^
	Very urgent	2.60	1.20			44.37	9.80		
	Uncertain	2.70	1.78			45.45	7.02		
	Do not know	3.04	2.49			45.09	3.92		
Impact of COVID-19 on mental health	Largely affected	2.67	0.97	8.174	0.017^b^	46.68	9.76	8.313	<0.016^b^
	Partially affected	2.49	1.56			43.78	7.76		
	No effect	3.88	2.58			46.22	7.22		

^a^Independent-sample t-test.

^b^one-way ANOVA.

SD: standard deviation; STAI-I: State-Trait Anxiety Inventory-I.

**Table 3:** Correlation between State‐Trait Anxiety Inventory, COVID-19 Knowledge Scale, and COVID-19-related related fear scores of women.

Scales	COVID-19 Knowledge Scale		Feeling herself in the high-risk group		Fear of COVID-19 transmission due to hospitalization		STAI	
Mean (±SD)	2.69 (1.58)		5.65 (2.87)		7.39 (3.06)		44.96 (8.48)	
	r	p^a^	r	p^a^	r	p^a^	r	p^a^
STAI-I	0.102	0.171	0.109	0.136	0.027^a^	0.712		
Feeling herself in the high-risk group	-0.036	0.635			0.437^a^	<0.001	0.109	0.136
Fear of COVID-19 transmission due to hospitalization	-0.090	0.228	0.437^a^	<0.001^b^			0.027^a^	0.712

^a^Pearson correlation test.

^b^Correlation is significant at the 0.01 level.

SD: standard deviation; STAI-I: State-Trait Anxiety Inventory-I.

## DISCUSSION

In this study, majority of women who had undergone gynecological surgery experienced fear of infection. Increased fear of COVID-19 transmission experienced by the majority of women in this study, which has been reported even in non-risk groups, is an expected result due to the increased risks associated with the surgical operation.

In our study, the most common concerns regarding the surgical process were being infected with the virus and uncertainty. The majority of the women perceived their surgical process as very urgent. The majority of the women felt fear mostly for being infected with virus. A study conducted to determine the implications of COVID-19 on women with gynecologic cancer reported that women were afraid of getting COVID-19 infection from the hospital setting while receiving treatment or follow-up. Besides, the same study indicated that women were concerned about the progression of the disease due to postponing treatment or follow-up^1^. In line with the data obtained from our study and the limited number of studies conducted, it has been determined that treatments requiring hospitalization and surgical procedures worry the patients^1,15,16,17^.

Studies on different female populations to evaluate COVID-19-related fear and anxiety have stated that women are afraid of infection and have high anxiety levels^1,15,16,18^. Similarly, increased rates of anxiety and depression have been reported in studies conducted on women with gynecological cancer^1,7^. Parallelly, our study demonstrated that the anxiety levels of women who had undergone gynecological surgery were higher.

Our study showed that married women experienced higher anxiety levels than single women (p<0.05). A study on patients with gynecological cancer found no relationship between the anxiety levels of women living alone and women not living alone^7^. Unlike these results, another study on patients in the preoperative period who were scheduled for elective surgery during the pandemic found that single patients experienced higher levels of anxiety^17^. In this study, it was estimated that the reason for the anxiety experienced by married women might be the decrease in social support due to visitor restrictions or the concerns of women about the transmission of COVID-19 to their cohabitants. A study evaluating the impact of visitor restriction rules on the postoperative experience of COVID-19-negative patients undergoing surgery reported no difference between groups with and without visitor restriction in terms of anxiety levels; however, the same study found that the visitor restriction group experienced social isolation due to reduced social support^19^. Decreased social support in the postoperative period causes patients to be mentally affected. This study demonstrated low education level causes increased anxiety in women undergoing gynecological operations. This study’s results also showed that women who paid attention to warnings about COVID-19 had higher anxiety scores (p<0.05). It is thought that increased sensitivity related to COVID-19 infection increases anxiety.

In our study, more than half of the women considered the urgency of their gynecological operation to be very urgent. The anxiety levels of women who perceived the urgency of their surgical operation to be very urgent were found to be higher (p<0.05). Studies in the literature have reported that 96–98% of surgical operations performed during the pandemic are elective surgery^9,20^. It is important to evaluate the perceptions of women about the surgical procedures they had undergone and to inform them about this issue to reduce the level of anxiety experienced by them.

In this study, the COVID-19 knowledge scores of retired women were lower than the general average (p<0.05). This result is thought to be due to the higher mean age of retired women. It is recommended that retired women should be given more detailed information about COVID-19.

The COVID-19 knowledge scores of women who had undergone cancer surgery were higher than other women. Since cancer patients are in the risk group for COVID-19, it is assumed that the knowledge level of women in this group is higher. Studies in the literature have reported increased anxiety levels in women with cancer^1,7,21,22^.

According to our results, women had high anxiety scores and very low COVID-19 knowledge. Besides, women had moderate levels of feeling in the high-risk group and experienced higher coronavirus transmission fear. A study conducted in the US (2020) reported that adults believed to be at high risk for severe COVID-19 delayed or avoided their urgent or routine medical care^2^. It should be taken into account that the feelings of women in the high-risk group may be a barrier to visiting for follow-up in the postoperative period. Therefore, there is a need for studies evaluating patient outcomes in the late postoperative period.

It is well known that regarding the pandemic, the prevalence of general gynecologic physical exams was reduced among women due to the fear of virus transmission^23^. A study on gynecological cancer patients found that most women considered themselves in the risk group for COVID-19 transmission^1^. We found that as the level of regarding themselves in the risk group for COVID-19 transmission increased, women’s fear of being infected with COVID-19 due to hospitalization increased. Accordingly, we believed that consideration of being in the risk group may be an obstacle for women to present to the hospital.

## CONCLUSION

This study demonstrated that women were afraid of COVID-19 infection during gynecological operation, the anxiety levels of women who perceived the urgency of the surgical operation they had undergone to be very urgent were higher, and as the level of considering themselves in the risk group for COVID-19 transmission increased. Therefore, especially healthcare personnel should inform women before gynecological operations, and interventions should be planned to reduce their anxiety. Considering that gynecological surgery can affect women’s lives in many ways, it is assumed that the problems experienced due to the pandemic may also reduce their quality of life. More studies are needed to determine other problems experienced by women undergoing gynecological surgery during the pandemic.
